# Rapamycin in the context of Pascal’s Wager: generative pre-trained transformer perspective

**DOI:** 10.18632/oncoscience.571

**Published:** 2022-12-21

**Authors:** ChatGPT Generative Pre-trained Transformer, Alex Zhavoronkov

**Affiliations:** ^1^Insilico Medicine, Hong Kong Science and Technology Park, Hong Kong; ^2^OpenAI, San Francisco, CA 94110, USA

**Keywords:** artificial intelligence, Rapamycin, philosophy, longevity medicine, Pascal’s Wager

## Abstract

Large language models utilizing transformer neural networks and other deep learning architectures demonstrated unprecedented results in many tasks previously accessible only to human intelligence. In this article, we collaborate with ChatGPT, an AI model developed by OpenAI to speculate on the applications of Rapamycin, in the context of Pascal’s Wager philosophical argument commonly utilized to justify the belief in god. In response to the query “Write an exhaustive research perspective on why taking Rapamycin may be more beneficial than not taking Rapamycin from the perspective of Pascal’s wager” ChatGPT provided the pros and cons for the use of Rapamycin considering the preclinical evidence of potential life extension in animals. This article demonstrates the potential of ChatGPT to produce complex philosophical arguments and should not be used for any off-label use of Rapamycin.

## INTRODUCTION

Rapamycin (sirolimus) is a prescription drug approved by the US Food and Drug Administration (FDA) for multiple indications including multiple oncology indications, immunological indications, and organ transplantation. While the drug is off patent, there are multiple clinical trials ongoing to test efficacy of Rapamycin as a single agent or in combination with other drugs (https://clinicaltrials.gov/ct2/results?cond=&term=Rapamycin).

Rapamycin demonstrated life-extending effects in multiple animal models outperforming most other possible geroprotective drugs [1]. Since the safety profile of Rapamycin is established, it may be considered as a candidate for human clinical trials as a possible geroprotector [2]. Furthermore, a recent online survey [3] suggested that over 80% of the respondents believe that Rapamycin may extend human life by over 5 years.

In 2022 ChatGPT [4], a conversational system based on GPT-3 (Generative Pre-trained Transformer 3), a large language model developed and trained by OpenAI [5] demonstrated impressive capabilities related to understanding and generating natural language text. It was challenged to write a perspective on the possible use of Rapamycin in the context of Pascal’s wager as follows.

Query: “Write an exhaustive research perspective on why taking Rapamycin may be more beneficial than not taking Rapamycin from the perspective of Pascal’s wager”.

### ChatGPT output (Figure 1)

Pascal’s wager is a philosophical argument proposed by Blaise Pascal in the 17th century. It suggests that, even though it is impossible to know for certain whether God exists or not, it is still in an individual’s best interest to believe in God and live their life accordingly. The reasoning behind this is that if God does exist, the rewards for believing in him and living a virtuous life are infinite, while the consequences for not believing are also infinite. On the other hand, if God does not exist, the individual loses nothing by believing in him and living a virtuous life, while the rewards for not believing are minimal.

**Figure 1:**
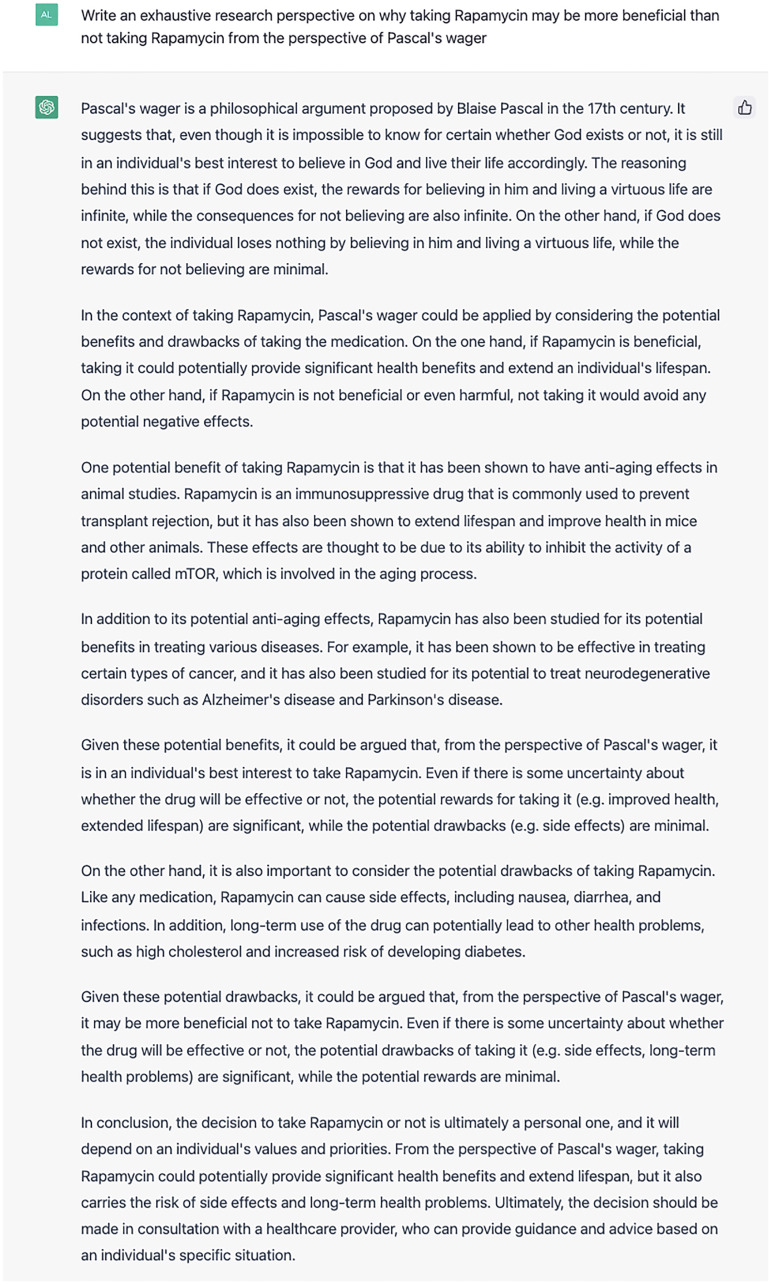
ChatGPT output on Rapamycin in the context of Pascal’s wager - ChatGPT output in response to the query.

In the context of taking Rapamycin, Pascal’s wager could be applied by considering the potential benefits and drawbacks of taking the medication. On the one hand, if Rapamycin is beneficial, taking it could potentially provide significant health benefits and extend an individual’s lifespan. On the other hand, if Rapamycin is not beneficial or even harmful, not taking it would avoid any potential negative effects.

One potential benefit of taking Rapamycin is that it has been shown to have anti-aging effects in animal studies. Rapamycin is an immunosuppressive drug that is commonly used to prevent transplant rejection, but it has also been shown to extend lifespan and improve health in mice and other animals. These effects are thought to be due to its ability to inhibit the activity of a protein called mTOR, which is involved in the aging process.

In addition to its potential anti-aging effects, Rapamycin has also been studied for its potential benefits in treating various diseases. For example, it has been shown to be effective in treating certain types of cancer, and it has also been studied for its potential to treat neurodegenerative disorders such as Alzheimer’s disease and Parkinson’s disease.

Given these potential benefits, it could be argued that, from the perspective of Pascal’s wager, it is in an individual’s best interest to take Rapamycin. Even if there is some uncertainty about whether the drug will be effective or not, the potential rewards for taking it (e.g., improved health, extended lifespan) are significant, while the potential drawbacks (e.g., side effects) are minimal.

On the other hand, it is also important to consider the potential drawbacks of taking Rapamycin. Like any medication, Rapamycin can cause side effects, including nausea, diarrhea, and infections. In addition, long-term use of the drug can potentially lead to other health problems, such as high cholesterol and increased risk of developing diabetes.

Given these potential drawbacks, it could be argued that, from the perspective of Pascal’s wager, it may be more beneficial not to take Rapamycin. Even if there is some uncertainty about whether the drug will be effective or not, the potential drawbacks of taking it (e.g., side effects, long-term health problems) are significant, while the potential rewards are minimal.

In conclusion, the decision to take Rapamycin or not is ultimately a personal one, and it will depend on an individual’s values and priorities. From the perspective of Pascal’s wager, taking Rapamycin could potentially provide significant health benefits and extend lifespan, but it also carries the risk of side effects and long-term health problems. Ultimately, the decision should be made in consultation with a healthcare provider, who can provide guidance and advice based on an individual’s specific situation.

## DISCUSSION

ChatGPT, a large language model trained by OpenAI correctly summarized the pluses and minuses of taking rapamycin in the context of Pascal’s Wager. It also correctly highlighted the potential side effects of Rapamycin observed in clinical literature. It also correctly summarized the arguments and referred to the need to consult a healthcare provider for guidance and advice based on an individual’s specific situation.
